# Multiscale spatial patterns of species diversity and biomass together with their correlations along geographical gradients in subalpine meadows

**DOI:** 10.1371/journal.pone.0211560

**Published:** 2019-02-27

**Authors:** Manhou Xu, Shixiong Zhang, Jing Wen, Xiaoyan Yang

**Affiliations:** Institute of Geographical Science, Taiyuan Normal University, Jinzhong, China; CNRS - Universite de Pau et des Pays de l'Adour - E2S UPPA, FRANCE

## Abstract

Researchers frequently discuss spatial distribution patterns of species diversity and biomass together with their correlations along geographical gradients. Typical subalpine meadows occur widely on the east of the Loess Plateau, China; here, we selected nine mountains belonging to four mountain systems from north to south on the east of the plateau. We analyzed five latitudinal and longitudinal gradients together with six elevational gradients to study the spatial distribution patterns of species diversity (including *α*, *β*, and *γ* diversity) and biomass plus with their relationships at various scales. Results showed that (1) for diversity, *α*-Diversity manifested unimodal variation patterns in horizontal spaces, peaking at high latitude and low longitude. However, *α*-diversity was not sensitive to elevation in vertical spaces and tended to decrease with increasing elevation. With increased latitude, longitude, and elevation, *β*-Diversity diminished; meanwhile, the rate of species turnover decreased and the similarity of community composition enlarged. *γ*-Diversity demonstrated quadratic function changes that were initially incremental and then decreased with increasing longitude, elevation, and latitude from 37.5° to 40°. In general, *β-*diversity had positive correlation with *γ*-diversity and negative correlation with *α-*diversity, which conformed to the function of *β* = *γ*/*α*. (2) For biomass, changes of aboveground biomass (AB) were more obvious along latitudinal gradients, whereas variations of belowground biomass (BB) had smaller differences along longitudinal and latitudinal gradients. More biomass was allocated to BB toward the north and east, whereas root-to-shoot ratio (R/S) was more evident at greater latitude than greater longitude. With increased elevation, more biomass was also allocated to BB, and the relationship of biomass to elevation was closer in AB. In short, the relation of biomass allocation tended to belowground plant parts with different geographical scales. (3) Species diversity had the strongest positive influence on AB. The Patrick and Shannon indices had correlations of power functions with AB and R/S, respectively, indicating that an allometric model could be used to model relationships between species diversity and biomass. In conclusion, the unique geomorphological structures with a series of basins between mountain systems on the east of the Loess Plateau, meant that subalpine meadows were mostly distributed along latitudinal directions, so the spatial distribution of species diversity and biomass was more evident along latitudinal gradients, and thus the response of aboveground biomass was more sensitive to variations of spatial gradients and species diversity.

## Introduction

Plants serve vital roles in terrestrial ecosystems and provide humans with many ecological services in regulating climate, improving soil fertility, protecting biodiversity, and promoting productivity [1–4]. Studies of species diversity and plant biomass have become important research topics related to ecology and geography, focusing on their spatial distribution and correlations along geographical gradients.

Biodiversity contributes significantly to sustaining a global ecological balance and to promoting sustainable development for humans [5–7]. As a measurable index of a community, biodiversity reflects essential features of ecosystems, represents variations that occur in ecosystem, and helps to maintain ecosystem productivity. Biodiversity is produced by competition among species in a community or in the processes involved in coordinating resources so that various species can coexist [8–10]; as a result, biodiversity provides provenance bases and supporting conditions for the operation and turnover of ecosystem functions [11–14]. Species diversity is a manifestation of biodiversity at the species level and is an important indicator that can be used to quantify and document community structure and composition. Species diversity reveals the organizational levels of a community and induces changes in the functional characteristics of a biotic community; it can even alter a shortage of critical species in a community or the utilization patterns of environmental resources by species, and thus lead to modifications in ecosystem structure and function [7, 15–18]. Variations in species diversity mirror changes of species richness and evenness in a community or habitat, as well as the relationships between a community and different natural geographical conditions [19]. In general, species diversity of plant community decreases from lower to higher latitude; in terms of elevation gradient, changes in species diversity of plant community have 5 models: negative correlation, greatest in mid-altitude, smaller in mid-altitude, positive correlation, and no correlation [16]. Thereby, studying and measuring species diversity is important and helps researchers to probe its patterns of variation along geographical gradients in modern studies of biodiversity.

Measurements of species diversity are primarily conducted at three spatial scales known as *α*-, *β*-, and *γ*-diversity [20–22]. The first scale is within-habitat diversity, that is, *α*-diversity, which mainly focuses on species number in local homogeneous habitat. At this scale, the principle factors that affect diversity are ecological niche diversity and interspecific interaction, so *α*-diversity is closely related to environmental energy [20, 21]. The second scale is between-habitat diversity, that is, *β*-diversity, which indicates differences of species composition among different habitats and communities or differences in turnover rates of species along environmental gradients. The dominant ecological factors that control *β*-diversity are soil, landform, and disturbance [20]. The last scale is regional diversity, that is, *γ*-diversity, which describes species number at regional or continental scales. The ecological processes that drive *γ*-diversity chiefly include hydrothermal dynamics, climate, as well as species development and evolution [21, 22]. Among these three types of diversity, *α*- and *β*-diversity constitute the overall diversity of communities or ecosystems, or habitat heterogeneity of a certain district.

Similar to species diversity, biomass is also a primary quantitive characteristic of ecosystems and reflects plants productivity; thus species diversity is a basic part of the study ecosystem function [16]. Biomass allocation among various organs mirrors the growth strategy a plant uses to adapt to an environment and plays a crucial role in the growth of plant individuals, species coexistence, and vegetation recovery [23, 24]. Strategies of biomass allocation among leaves, stems, and roots, together with allometric relationships between plant organs provide a foundation in the study of species evolution, maintenance of diversity, and carbon cycling in ecosystems, and also are important to our understanding of the distribution of carbon in ecosystems and the function of carbon sinks [25–27]. Biomass allocation, especially the allocated models under the effects of different geographical gradients, is important in studies of biomass. In principle, plant community biomass possesses obvious latitudinal and altitudinal variations [24, 26]. There are mainly 3 models with altitudinal gradient: negative correlation, positive correlation, and unimodal curve correlation; however, relatively less studies on the horizontal spatial distribution of plant community biomass [25, 27]. Therefore, further studies need to be carried out on spatial variations of plant community biomass with geographical gradients.

Soil erosion is one of the most important environmental issues affecting terrestrial ecosystems. In arid and semi-arid areas, runoff and erosion can reduce soil water-holding capacity and hinder the recovery of degraded ecosystems [28]. The Loess Plateau of China is a typical area suffering land degradation due to erosion, while vegetation restoration is the most effective biological tool to solve ecosystem degradation on the Plateau [28, 29]. Vegetation can provide better erosion control services when there is greater diversity and biomass and its spatial distribution also plays a crucial role in reducing water and soil losses at the slope scale [29, 30]. Subalpine meadows are one grassland type of the Loess Plateau and mainly are distributed in high-elevation mountains where their species diversity and biomass is obviously affected by the mountainous terrain [28]. Latitude, longitude, and elevation are dominant terrain indicators of mountainous subalpine meadows; they directly affect the spatial distribution of solar radiation and rainfall, and thus they result in an uneven distribution of soil moisture and temperature [29, 30]. Large areas of subalpine meadows on the east of the Loess Plateau have an abundance of species. These meadows not only provide excellent natural pastures but also serve as famous eco-attractions—for example, Heyeping has been honored as the “plateau jade,” Shunwangping as the “Jiuzhaigou of north China,” and Wutai Mountain as “the roof of north China.” With a rapid development of tourism and pasture husbandry, subalpine meadows have experienced extensive and severe degradation caused by humans in the mountain systems of Liuleng, Wutai, Lvliang, and Zhongtiao, where their environments are sensitive and fragile, meadows degradation had been increasing, and biodiversity has been threatened seriously [31, 32].

From a level of plant population in natural conditions, species diversity has important significance to the discussion of spatial distributions and correlations of species diversity and biomass at various levels; this illuminates the internal mechanisms of functional relationships between biodiversity and ecosystems. Given this, typical subalpine meadows were used as research objects on the east of the Loess Plateau. These ecosystems were divided into different latitudinal, longitudinal, and elevational belts, and then the following three scientific problems were addressed: (1) spatial distribution patterns of species diversity at the three scales of *α*, *β*, and *γ* diversities in subalpine meadows; (2) patterns of variation in biomass and biomass allocation at horizontal and elevational scales in subalpine meadows; and (3) correlations between species diversity and biomass in subalpine meadows.

## Materials and methods

### Study area

The Loess Plateau distributes the largest area of loess in the world and is characterized by rare precipitation, intense evaporation, severe soil erosion, and a low ability to resist natural hazards, so its ecological environment is harsh in nature and extremely difficult to recover when destroyed [31–33]. The east of the Plateau (34°34'–40°43' N, 110°14'–114°33' E) lies in Shanxi Province and serves as a dividing line between the second and third steps of topography in China and where subalpine meadows are distributed most intensively in the Plateau. Owing to complex and changeable topography, it develops Taihang mountain, Lvliang mountain, and a series of basins between them. From north to south, these basins are successively Datong, Xinding, Taiyuan, Changzhi, Linfen, and Yuncheng basins. The temperate continental monsoon climate features an annual mean temperature of 4–14°C, summer mean temperature of 22–27°C, winter mean temperature of −12 to −2°C, annual precipitation of 400–600 mm, and a frost-free season of 4–7 months [31]. Vegetation in this region dominates as the vegetation type of temperate regions with complicated geomorphic features and combinations of water and heat. Mountainous areas occupy more than 80% in this region; among larger mountain systems, subalpine meadows cover an area of about 353,000 hm^2^; these are mainly distributed in high-elevation belts above the timberline in the Liuleng, Lvliang, Wutai, and Zhongtiao mountain systems. Plants in subalpine meadows principally include perennial herbs that are suitable for low temperature and moderate moisture; for example, *Carex*, *Kobresia*, Asteraceae, Leguminosae, and other cyperaceous species were common [32]. The soil in subalpine meadows is high organic content and has thick litter layer.

### Experimental design

**Fig 1:**
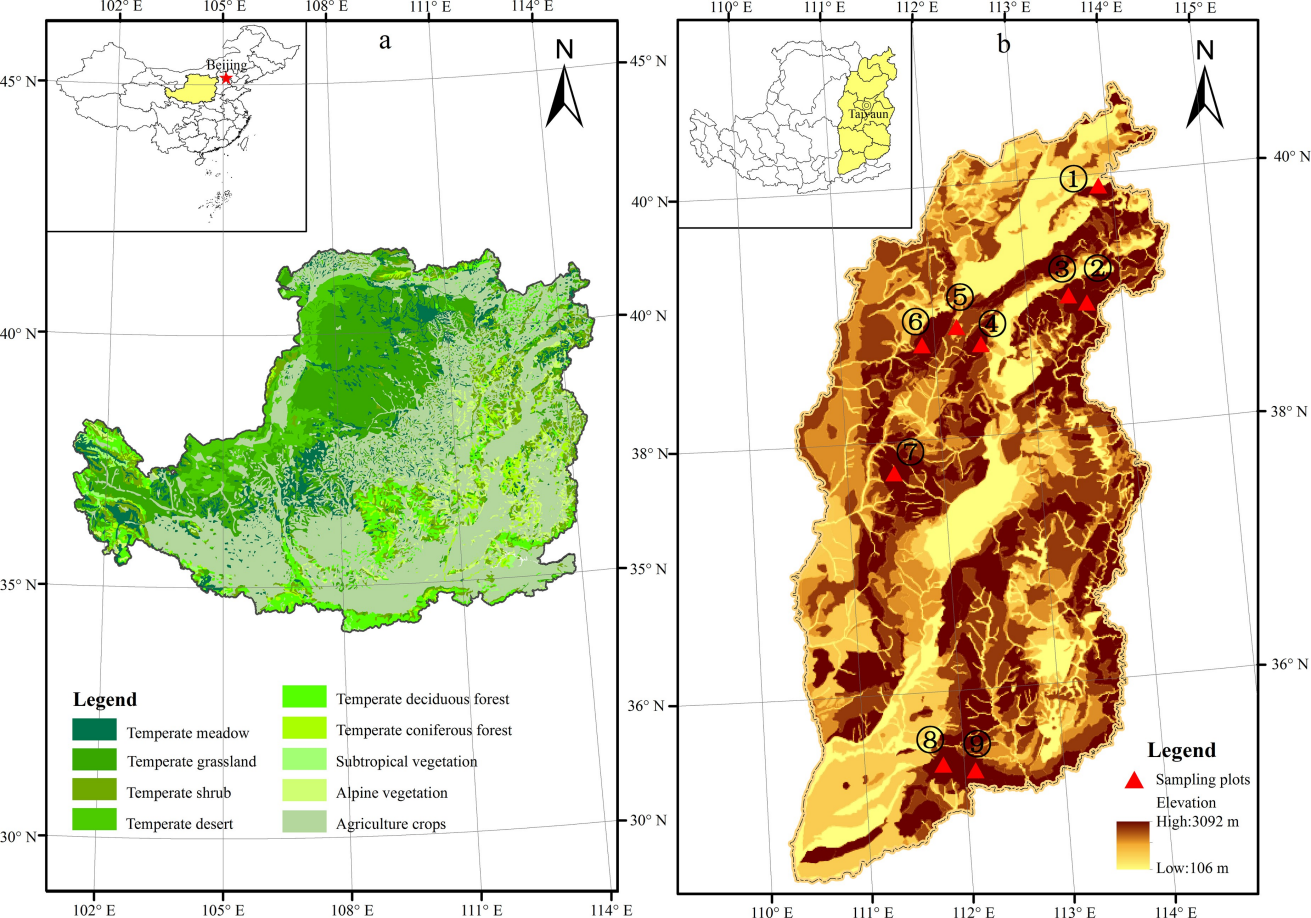
Study area and experimental plots. Light and dark colors show low and high elevation, respectively.

Comparing with a vegetation-type map of the Loess Plateau (Fig 1A) and a topographic map of Shanxi Province (Fig 1B), experimental plots were selected in typical meadows of subalpine belts at higher elevation in mountains along the east of the Loess Plateau. These were investigated from July to August in 2016 in areas with little human disturbance, flat terrain, and a uniform distribution of vegetation. On the entire east, nine subalpine meadows (one subalpine meadow in each mountain) were successively surveyed in different mountain ranges moving from north to south. In total, nine mountains belonged to four mountain systems were surveyed using plots. The names of these mountains were Dianding (DD) Mountain in the Liuleng mountain system, Beitai (BT) and Dongtai (DT) mountains in Wutai mountain system, Malun (ML), Heyeping (HY), Yunzhong (YZ) and Yunding (YD) mountains in the Lvliang mountain system, and Shunwangping (SU) and Shengwangping (SE) mountains in the Zhongtiao mountain system (Fig 1B). In the whole mountain systems, DD, BT and DT were geographically classified as northern mountains, ML, HY, YZ and YD as central mountains, and SU and SE as southern mountains from north to south. Their geographic coordinates for the 9 mountain sites surveyed were listed in Table 1.

**Table 1:** Geographical data and abbreviations for nine mountains surveyed on the east of the Loess Plateau with two, four, and two mountains in its northern, central, and southern parts, respectively.

Location	Number	Mountain name/abbreviation	Latitude/°N	Longitude/°E	Elevation/m
Northern part	①	Dianding (DD)	39.85	113.94	2265
	②	Dongtai (DT)	39.05	113.67	2565
	③	Beitai (BT)	39.08	113.57	3045
Central part	④	Yunzhong (YZ)	38.68	112.43	2260
	⑤	Malun (ML)	38.75	111.93	2710
	⑥	Heyeping (HY)	38.71	111.84	2745
	⑦	Yunding (YD)	37.88	111.54	2690
Southern part	⑧	Shunwangping (SU)	35.42	111.96	2250
	⑨	Shengwangping (SE)	35.34	112.21	1720

Additionally, prior to carrying out this experiment, we obtained permissions from the Luyashan National Nature Reserve (Xinzhou city) for DD, BT and DT; from the Pangquangou National Nature Reserve (Lvliang city) for ML, HY, YZ and YD; and from the Wulushan National Nature Reserve (Linfen city) for SU and SE. We successfully obtained these permissions as our field studies did not involve endangered or protected species.

### Measurement of plant diversity

On each of these nine mountains, six 1 m^2^ plots were installed randomly to survey plant diversity at a community scale or a total of 54 plots on all mountains. We used 1 m × 1 m quadrat frames as measurement tools, which we divided into 100 uniform grids (0.1 m × 0.1 m). In each grid, we measured plant height, abundance, coverage, and frequency of each species; the data were used to calculate species diversity indices in the plots (see 1.5 Data analysis). Meanwhile, we recorded the latitude, longitude, and elevation of each plot by a portable GPS (Table 1). Using the horizontal directions of latitude and longitude, the nine study plots were divided into five latitudinal and five longitudinal gradient belts with 0.5° and 0.45° intervals arranged from south to north and from west to east, respectively. The nine plots were divided into six elevational gradient belts from low to high elevation with an interval of 100 m (Table 2).

**Table 2:** Demarcations on geographical gradient belts of various mountains. Latitudinal, longitudinal, and elevational gradients were divided into five, five, and six belts with intervals of 0.5°, 0.45°, and 100 m, respectively. The initial belts were all numbered 1 with different gradient ranges. Mountain names indicated by abbreviated letters are shown in Table 1.

Geographical gradient belt	Belt number	Geographical gradient range	Geographical gradient	Mountain name
Latitudinal belt (0.5° intervals)/°N	1	35–35.5	35.34	SE
			35.42	SU
	2	37.5–38	37.88	YD
	3	38.5–39	38.68	YZ
			38.71	HY
			38.75	ML
	4	39–39.5	39.05	DT
			39.08	BT
	5	39.5–40	39.85	DD
Longitudinal belt (0.45° intervals)/°E	1	111.15–111.6	111.54	YD
	2	111.6–112.05	111.84	HY
			111.93	ML
			111.96	SU
	3	112.05–112.5	112.21	SE
			112.43	YZ
	4	113.4–113.85	113.57	BT
			113.67	DT
	5	113.85–114.3	113.94	DD
Elevational belt (100 m intervals)/m	1	1700–1800	1720	SE
	2	2200–2300	2250	SU
			2260	YZ
			2265	DD
	3	2500–2600	2565	DT
	4	2600–2700	2690	YD
	5	2700–2800	2710	ML
			2745	HY
	6	3000–3100	3045	BT

Note: Table 1 lists the full mountain names and abbreviations.

### Measurement of plant biomass

At each of nine mountain sites, we randomly located five plots for use in measuring diversity, with 45 plots in total. A 0.2 m × 0.2 m quadrat was placed in the center of each plot with its plants being evenly distributed, so that 45 quadrats were also acquired in total. We used quadrats to survey plant biomass at the community scale using the following method. In each quadrat, aboveground plant parts were clipped near the ground surface and belowground plant parts were then excavated in the entire 0.2 m × 0.2 m quadrat to a depth of 0.2 m. Samples of aboveground plant parts and soil blocks with volumes of 0.2 m × 0.2 m × 0.2 m were sealed and brought to laboratory for post-processing. In this process, soil blocks and withered grass were removed from samples of aboveground plant parts and only live plants were retained. Samples of belowground plant parts were first sieved by a standard soil sieve with a bore diameter of 0.42 mm to eliminate stones and coarse debris from the soil. Then, the soil samples were sieved using a standard soil sieve with a bore diameter of 0.18 mm to eliminate fine roots with diameters no smaller than 0.18 mm. Next, live roots were separated from root systems according to color, suppleness, and whether or not hair roots were present. Live roots and treated stems and leaves were placed into an oven and dried for 48 h to constant weight at a high temperature of 80°C. Last, dry samples were weighed as below- and aboveground plant biomass by an electronic balance with an accuracy of 0.001 g.

### Data analysis

#### (1) *α*-Diversity

We calculated the important species values based on the relative height, abundance, coverage, and frequency of each species; then *α*-diversity indices were obtained, including the Simpson, Shannon, and Pielou indices. *α*-Diversity indices plus the richness index of Patrick were used to explore the characteristics of spatial distribution of subalpine meadows at the scale of *α*-diversity along with changes in latitude, longitude, and elevation. Corresponding computational formulas of these *α*-diversity indices were as follows [33]:

$$IV=\frac{rh+ra+rc+rf}{4},$$ (1)

R=S, (2)

$$H^{\prime }=1-\overset{S}{\underset{i=1}{\sum }}p_{i}^{2},$$ (3)

$$H=-\overset{S}{\underset{i=1}{\sum }}p_{i}\ln\left(p_{i}\right),$$ (4)

$$E=\frac{H}{\ln(S)},\;\mathrm{and}$$ (5)

$$p_{i}=\frac{IV_{i}}{IV_{total}},$$ (6)

where *IV* is the importance value, *rh* is relative height, *ra* is relative abundance, *rc* is relative coverage, and *rf* is relative frequency; in addition, *R*, *H′*, *H*, and *E* are the Patrick, Simpson, Shannon, and Pielou indices, respectively; *i* is a plant species *i* and *S* are the sum of all plant species in the plots.

#### (2) *β*-Diversity

Species in different plots were merged in each geographical gradient belt and variations of *β*-diversity indices were analyzed between two adjacent gradient belts to probe the differentiation in the characteristics of subalpine meadows at the scale of *β*-diversity with latitude, longitude, and elevation. Measurements of *β*-diversity chiefly included two aspects: (1) community dissimilarity based on species composition and (2) species replacement based on the distribution boundary [34]. Unlike *α*-diversity, measurements of *β*-diversity could be separated into two methods: binary attribute data and quantitative data, respectively. Hence, we used two indices, the Cody and Sørenson indices, in the analysis of *β*-diversity based on binary attribute data; in addition, we used the Bray-Curtis index based on quantitative data. Corresponding computational formulas of these *β*-diversity indices are as follows:

$$\beta_{C}=\frac{a+b-2c}{2},$$ (7)

$$\beta_{S}=1-\frac{2c}{a+b},\;\mathrm{and}$$ (8)

$$\beta_{B‑C}=\frac{2cIV}{a+b},$$ (9)

where *β*_C_, *β*_S_, and *β*_B–C_ are the Cody, Sørenson, and Bray-Curtis indices, respectively; *a* and *b* are the number of species in plots A and B, respectively, and *c* is the number of common species between plots A and B; and *cIV* is the sum of the relatively small important values of species common to both A and B, that is, *cIV* = ∑min (*cIV*_*a*_, *cIV*_*b*_).

#### (3) *γ*-Diversity

*γ*-Diversity indicates species richness of all habitats within a certain geographical range and is usually used to demonstrate species number in a region or on a continent [34]. Here, regional scales were employed using five latitudinal and longitudinal gradients together with six elevational gradients; these regions were the divided into nine groups of subalpine meadows. Total species number (i.e., total species richness of *S*) in each geographic gradient belt was defined as an indicator to investigate spatial distribution patterns of subalpine meadows at the scale of *γ*-diversity.

#### (4) Relationship among *α*, *β*, and *γ* diversity

Firstly, *α* and *γ* diversity were recalculated basing on species number singly in each gradient belt. Then, common functions as β = γ/α, β = γ–α, and β = 1–α/γ [21, 35, 36] were adopted to calculate the simulated value of β-diversity. Finally, to probe the relationship among *α*, *β*, and *γ* diversity, correlation analyses were conducted in measured value of β-diversity with *α* and *γ* diversity, as well as with its simulated value.

#### (5) Biomass

Below- and aboveground biomass was added to calculate total biomass and the root-to-shoot ratio was also found for each unit. Therefore, aboveground biomass, belowground biomass, total biomass, and the root-to-shoot ratio were adopted as biomass indicators and were used to analyze variations of plant community biomass at different latitude, longitude, and elevation in subalpine meadows.

Lastly, OriginPro 9.1 software (Origin Lab, Northampton, MA) was used to draw spatial distributions of *α*, *β*, and *γ* diversity plus with variations of biomass with latitude, longitude, and altitude. SPSS 16.0 software (SPSS Inc., Chicago, IL, USA) was adopted to conduct regression analyses between *α*-diversity and biomass; correlation analyses among *α*, *β*, and *γ* diversity; together with correlation and significance analyses of *α*-diversity with elevation and among various mountains, respectively.

## Results

### Spatial distribution characteristics of species diversity in subalpine meadows

#### *α*-Diversity

**Fig 2:**
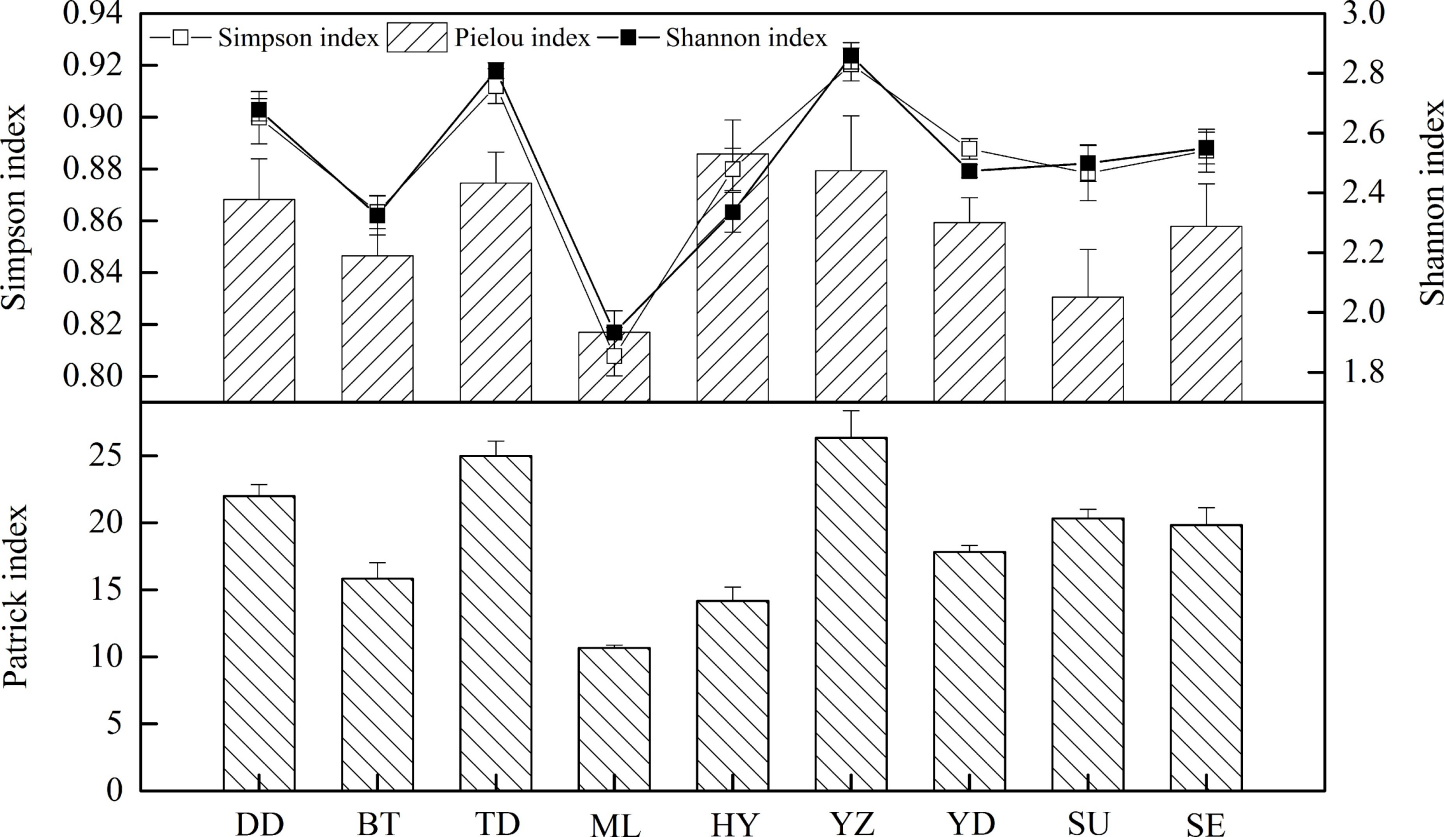
Spatial distribution of *α*-diversity in subalpine meadows. *α*-Diversity indices included Simpson, Shannon, Pielou, and Patrick indices. The data were collected from six quadrats on each of nine mountain sites (54 quadrats in total); means for each mountain site were used in the analysis. Therefore, each *α*-diversity index had nine values, one for each site.

We observed large differences in different mountain ranges for the Simpson (*H′*), Shannon (*H*), Pielou (*E*), and Patrick (*R*) indices in subalpine meadows. However, these *α*-diversity indices had relatively consistent trends that all presented variations in wave curves ranging from the northernmost DD to the southernmost SE (Fig 2). Maximums of *α*-diversity indices appeared in YZ (average, 7.75), and their minimum appeared in ML (average, 3.56). The maximums and minimums for the *α*-diversity indices all appeared in central mountains (from ML to YD) and their differences were significant (*P*<0.05), showing a greater fluctuation of *α*-diversity in the central mountains than elsewhere. iIn quantitative terms, *α*-diversity of the subalpine meadows was smallest in the central mountains, whereas it was greater in the northern (from DD to DT) and southern (from SU to SE) mountains with no larger differences between them. Except for *E*, changes were very consistent for *H′*, *H*, and *R*, and reached significant levels (*P*<0.05) between adjacent mountains from DD to YD; meanwhile, changes of *E* were significant (*P*<0.05) only between adjacent mountains from DT to HY. However, from YD to SE, the *α*-diversity indices had insignificant differences (*P*>0.05) (Table 3).

**Table 3:** Correlation and significance analyses of *α*-diversity with elevation and among various mountains, respectively. *α*-Diversity indices included the Patrick, Simpson, Shannon, and Pielou indices. Mountains were DD, BT, DT, ML, HY, YZ, YD, SU, and SE; mountain names indicated by abbreviated letters are shown in Table 1. Different or the same small letters present significant or insignificant differences (*P*<0.05 and *P*>0.05), respectively. The data for correlation coefficients in the analyses of *α*-diversity with elevation are listed with the corresponding *P* values in brackets. In significance analyses of *α*-diversity among various mountains, significance levels were expressed with different small letters. The data were collected from six quadrats on each of nine mountain sites (54 quadrats in total); means for each mountain site were used in the analysis. Therefore, each mountain site had one elevation, so nine *α*-diversity values were used in each correlation analysis. However, the six plots in each mountain were not averaged, so six value were used in each significance analysis for each site.

Analysis type	Items	Patrick Index	Simpson Index	Shannon Index	Pielou Index
Correlation	Elevation/m	−0.5 (*P* = 0.17)	−0.387 (*P* = 0.303)	−0.463 (*P* = 0.21)	−0.103 (*P* = 0.792)
Significance	DD	*bc*	*ab*	*bc*	*ab*
	BT	ef	c	e	*abc*
	DT	*ab*	*a*	*ab*	*ab*
	ML	g	d	f	*c*
	HY	f	*bc*	e	*a*
	YZ	a	a	a	*a*
	YD	de	*bc*	d	*abc*
	SU	*cd*	*bc*	*d*	*bc*
	SE	*cd*	*bc*	*cd*	*abc*

Note: Table 1 lists the full mountain names and abbreviations.

**Fig 3:**
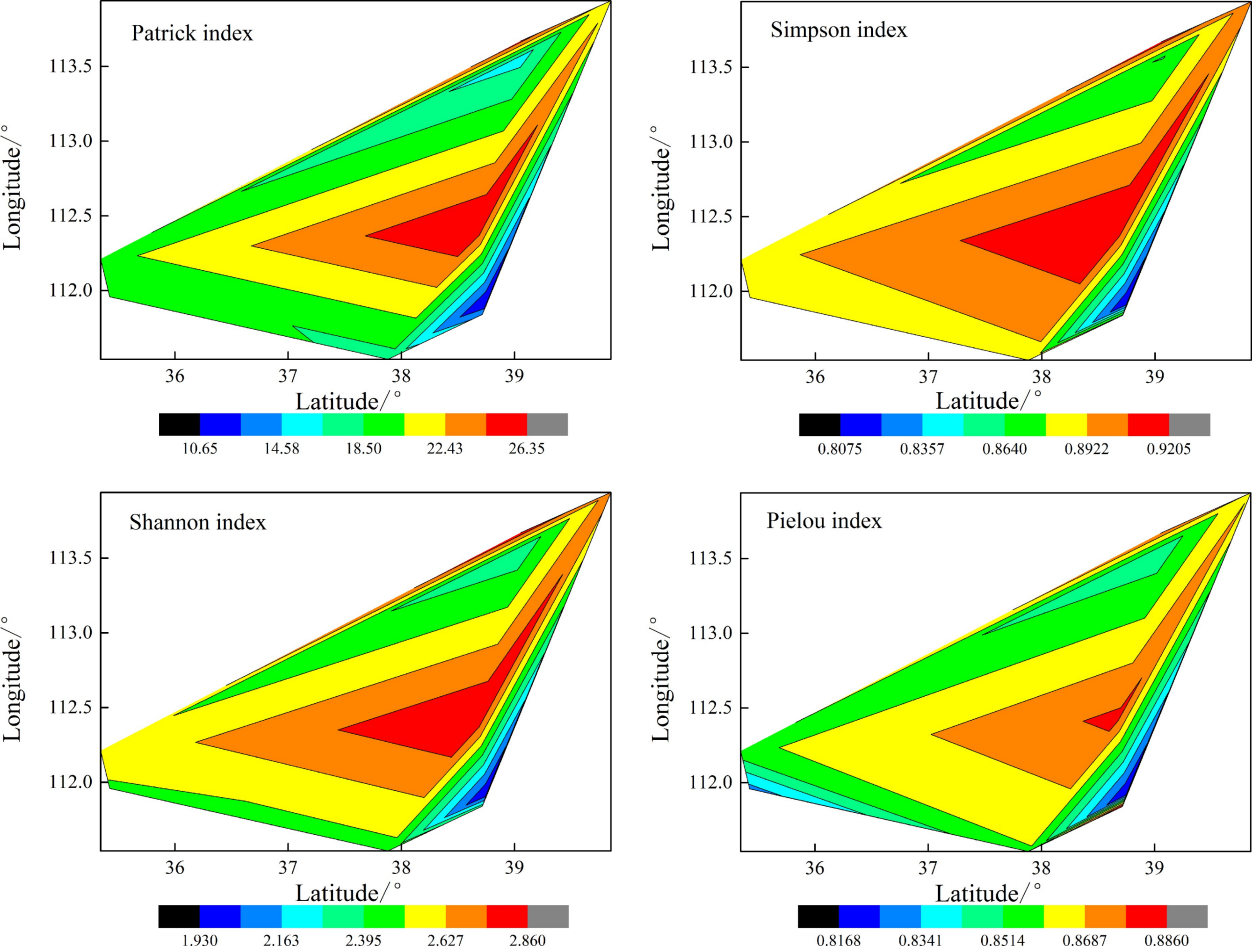
Variations of *α*-diversity with latitude and longitude in subalpine meadows. *α*-Diversity indices included the Patrick, Simpson, Shannon, and Pielou indices. The data were collected from six quadrats on each of nine mountain sites (54 quadrats in total) with different latitude and longitude; means for each mountain site were used in the analysis. Therefore, each index had nine values, one for each site.

In horizontal spaces, *α*-diversity indices of subalpine meadows showed similar patterns of change with latitude and longitude (Fig 3). With increasing latitude (from 35.34° to 39.85° N) and longitude (from 112.21° to 113.94° E), *R*, *H′*, *H*, and *E* tended to initially increase and then decrease, presenting unimodal curves with peak values being at relatively high latitudes (38.7° N) and relatively low longitudes (112.4° E), respectively. Maximums of *R*, *H′*, *H*, and *E*, which were 26.3, 0.92, 2.86, and 0.88, respectively, all existed near 38.7° N and 112.4° E. Trends of *α*-diversity indices had larger discrepancies with latitude and longitude at a latitudinal boundary of 38°. When latitude was below 38°, *α*-diversity indices changed more gently with latitude; with longitude, *α*-diversity indices experienced symmetrical changes with an axis of symmetry at 112.3°. When latitude was greater than 38°, *α*-diversity indices changed more dramatically with latitude; with longitude, *α*-diversity indices showed asymmetrical changes that was more dramatic initially and then less so. With changes in elevation, *R*, *H′*, *H*, and *E* had negative correlations with elevation (mean coefficients, −0.363), but correlations were all insignificant (*P*>0.05), demonstrating that *α*-diversity was affected insignificantly by elevation and tended to decrease with increasing elevation (Table 3).

#### *β*-Diversity

**Fig 4:**
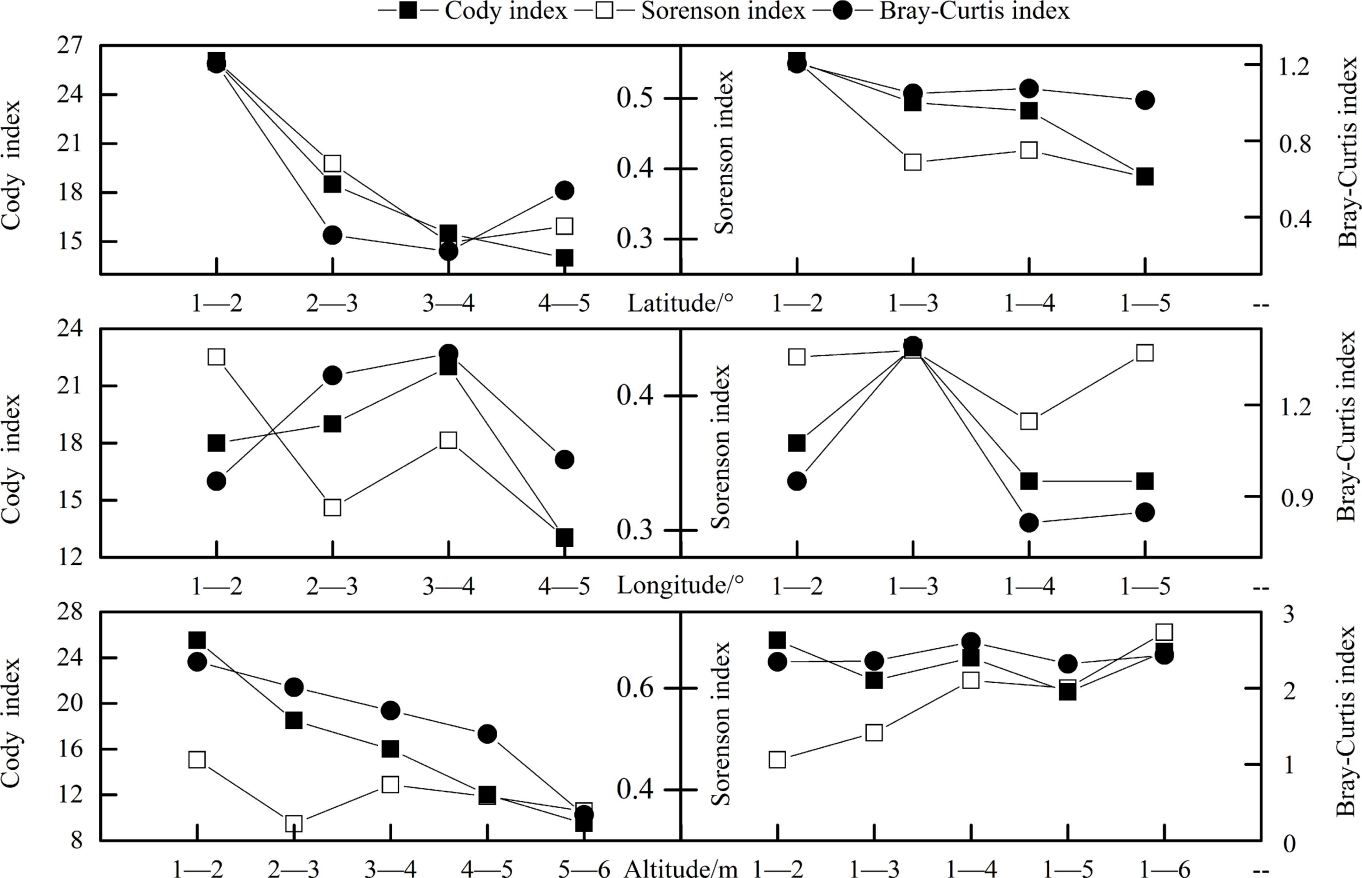
Changes on spatial distribution of *β*-diversity in subalpine meadows. *β*-Diversity indices included the Cody, Sørenson, and Bray-Curtis indices. Numbers from 1 to 6 in *x*-coordinates represented corresponding numbers of latitudinal, longitudinal, and elevational belts in Table 2. Intervals of 1–2, 2–3, 3–4, 4–5, and 5–6 indicated comparisons between adjacent belts, whereas 1–2, 1–3, 1–4, 1–5, and 1–6 indicated comparisons of the initial belt (belt 1) with other belts. The study employed five latitudinal, five longitudinal, and six elevational belts. Each index had values for four latitudinal, four longitudinal, and five elevational belts.

Various trends existed in *β*-diversity with changes in latitude, longitude, and elevation in subalpine meadows, but in the same type of gradient belts, more consistent trends were shown for Cody (*β*_C_), Sørenson (*β*_S_), and Bray-Curtis (*β*_B–C_) indices (Fig 4).

Along latitudinal gradients, relative to an initial belt (belt number 1), *β*_C_, *β*_S_, and *β*_B–C_ all decreased between different latitudinal belts with amplitudes of 26.9%, 29.8%, and 32.7%, respectively. Between adjacent latitudinal belts, *β*_C_, *β*_S_, and *β*_B–C_ exhibited decreased trends with amplitudes being obviously greater at 46.2%, 42.5%, and 62.8%, respectively. Overall, *β*_C_, *β*_S_, and *β*_B–C_ all declined along latitudinal gradients with mean amplitudes of 36.55%, 36.15%, and 47.75%, respectively.

Similarly, along longitudinal gradients, *β*_C_, *β*_S_, and *β*_B–C_ all decreased and their reduced amplitudes were −19.45%, −15.27%, and −0.75%, respectively; along elevational gradients,*β*_C_ and *β*_B–C_ declined with corresponding mean amplitudes of −33.33% and −29.85%, whereas *β*_S_ increased with its amplitude being 16.35%.

#### *γ*-Diversity

**Fig 5:**
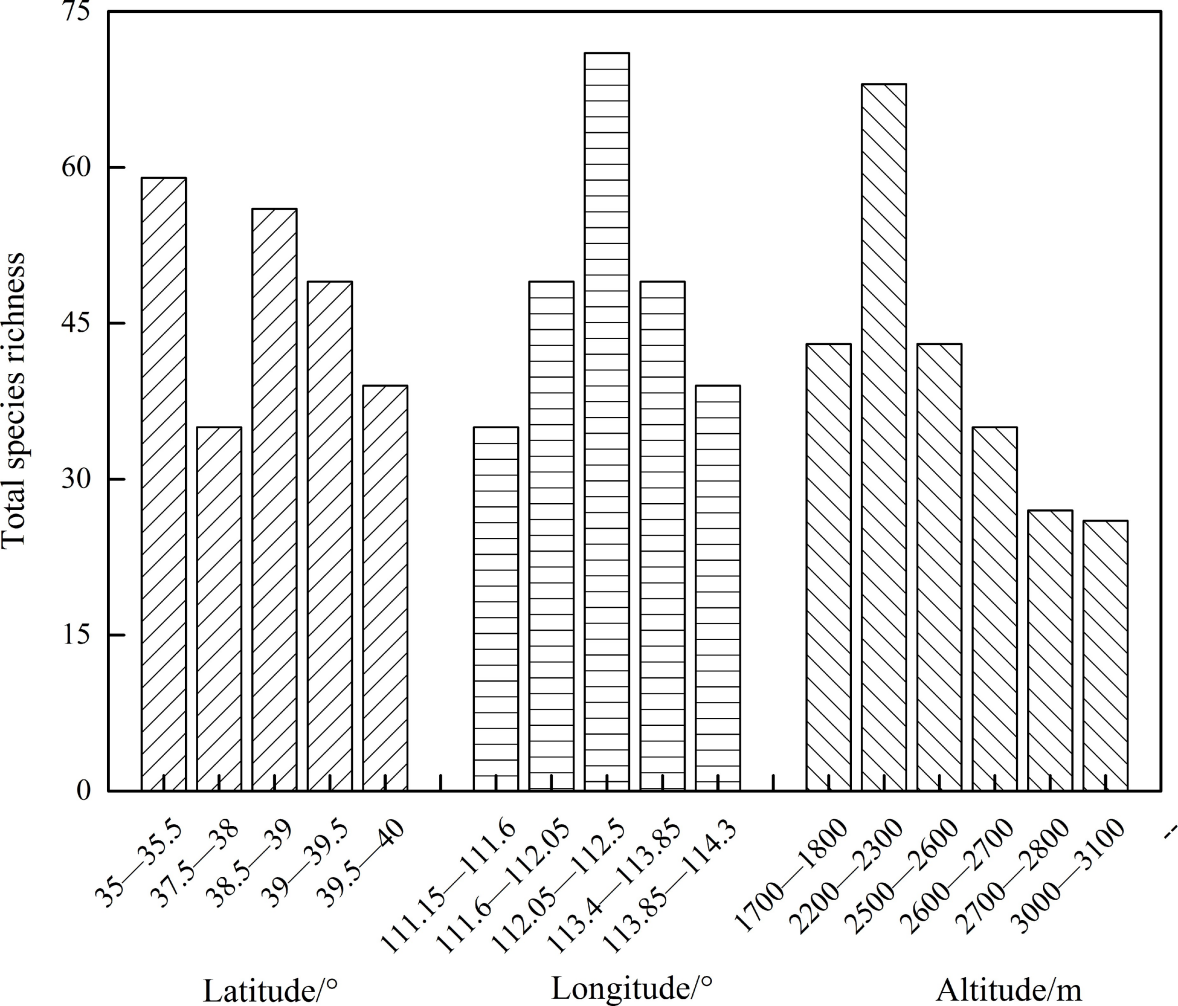
Spatial distribution of *γ*-diversity in subalpine meadows. The study employed corresponding belts along five latitudinal, five longitudinal, and six elevational gradients, with five, five, and six values respectively. Each belt had one value.

The spatial distribution of subalpine meadows was highly consistent at the scale of *γ*-diversity (Fig 5). Along latitudinal gradients, total species richness (*S*) generally exhibited a decreasing trend of a logarithmic function (*R*^2^ = 0.185, *P*>0.05), but it presented a significant variation of a quadratic function with a trend of initially increasing and then decreasing at latitudinal belts from 37.5° to 40° (*R*^2^ = 0.885, *P*<0.01). Along longitudinal gradients, *S* significantly demonstrated the variation of a quadratic function with a tendency of initially increasing and then decreasing (*R*^2^ = 0.784, *P*<0.01); its peak value appeared at longitudinal belts between112.05° and 112.5°. Along elevational gradients, *S* also showed a quadratic function variation with a trend of initially increasing and then decreasing (*R*^2^ = 0.598, *P*<0.05), while peaking at elevational belts between 2200 m and 2300 m. Averages of *S* along latitudinal, longitudinal, and elevational gradients were 47.6, 48.6, and 40.3, respectively, illustrating that species distribution was slightly greater in horizontal spaces than that with changes in elevation at regional scales.

#### Correlation among *α*, *β*, and *γ* diversity

*β*_C_ and *β*_S_ (measured values) were all calculated basing on binary attribute data and had similar correlation with *α-*diversity, *γ*-diversity, and their simulated values (Table 4). They had negative and positive correlations with *γ* and *α* diversity, respectively. Among negative correlations with their simulated values, the greatest correlation coefficients of *β*_C_ (0.851) and *β*_S_ (0.622) were all in the function of *β* = *γ*/*α*. While for *β*_B–C_ (measured value), it was calculated according to quantitative data and had corresponding positive and negative correlations with *γ* and *α* diversity, which was opposite to *β*_C_ and *β*_S_. Among positive correlations with its simulated value, the greatest correlation coefficient of *β*_B–C_ (0.932) also existed in the function of *β* = *γ*/*α*. Thereby, although a larger difference appeared in *β-*diversity with different data types, *β-*diversity had positive correlation with *γ*-diversity and negative correlation with *α-*diversity, which conformed to the function of *β* = *γ*/*α*.

**Table 4:** Correlation analyses among *α*, *β*, and *γ* diversity. *β*_C_ is Cody index, *β*_S_ is Sørenson index, and *β*_B–C_ is Bray-Curtis index in *β-*diversity. There are 7, 7 and 9 gradient belts with latitude, longitude and altitude, respectively. Simulated values of *β-*diversity are calculated with common functions of β = γ/α, β = γ–α, and β = 1–α/γ. *α* and *γ* diversity are recalculated basing on species number in each gradient belt. So 23 data are shown for each diversity. The data are correlation coefficients and the levels of correlation analyses are all significant (*P*<0.01).

Measured value of *β*-diversity	*γ*-diversity	*α*-diversity	Simulated value of *β*-diversity		
			*β* = *γ*/*α*	*β* = *γ*–*α*	*β* = 1–*α*/*γ*
*β*_C_	-0.841	0.853	-0.851	-0.849	-0.808
*β*_S_	-0.581	0.562	-0.622	-0.581	-0.534
*β*_B–C_	0.873	-0.923	0.932	0.890	0.823

### Spatial distribution characteristics of biomass in subalpine meadows

#### The fluctuation of biomass in various mountains

Biomass indices of subalpine meadows fluctuated in various mountains (Table 5). Corresponding coefficients of variation in aboveground biomass (AB), belowground biomass (BB), total biomass (TB), and the root-to-shoot ratio (R/S) were 0.614, 0.437, 0.373, and 0.668, demonstrating that the fluctuation in AB was larger than BB in subalpine meadows but was smaller for TB.

**Table 5:** Spatial distribution of biomass in subalpine meadows. Mountain names indicated by abbreviated letters are shown in Table 1. AB, BB, TB, and R/S represent aboveground, belowground, and total biomass, as well as the root:shoot ratio, respectively. Different or the same small letters indicate significant and insignificant differences (*P*<0.05 and *P*>0.05), respectively. The data are shown with mean ± S.E. with six values given for each mountain.

Mountain name	AB/(g/m^2^)	BB/(g/m^2^)	TB/(g/m^2^)	R/S
DD	126.400 ± 16.348 *d*	552.350 ± 139.573 *bc*	678.750 ± 149.120 *bcde*	4.268 ± 0.800 *bcd*
BT	85.550 ± 12.002 *d*	901.400±104.680a	986.950 ± 114.034 *ab*	10.880±1.041a
DT	125.100 ± 8.622 *d*	414.100 ± 42.257 *c*	539.200 ± 39.461 *cde*	3.417 ± 0.456 *bcd*
ML	72.100 ± 5.233 *d*	369.600 ± 51.519 *c*	441.700 ± 54.890 *de*	5.075 ± 0.626 *bc*
HY	235.250±29.123bc	787.850±55.681ab	1023.100±60.086a	3.675 ± 0.650 *bcd*
YZ	143.400 ± 33.474 *d*	258.050 ± 62.421 *c*	401.450 ± 65.011 *de*	2.202 ± 0.657 *cd*
YD	54.850 ± 6.957 *d*	310.050 ± 70.802 *c*	364.900 ± 68.983 *e*	6.412 ± 0.839 *b*
SU	293.150 ± 51.611 *ab*	415.600 ± 61.728 *c*	708.750 ± 89.694 *abcd*	1.638 ± 0.334 *d*
SE	344.950 ± 48.528 *a*	458.750 ± 106.270 *c*	803.700 ± 137.171 *abc*	1.337 ± 0.232 *d*

Note: AB, aboveground biomass; BB, belowground biomass; TB, total biomass; R/S, root: shoot ratio. Table 1 lists the full mountain names and abbreviations.

From the northernmost site, DD, to the southernmost, SE, AB was varied with a wave form and tended to increase (*R*^2^ = 0.402, *P*>0.05) with larger values existing in the southern sites of SE and SU as well as the central site of HY. The AB was significantly larger in southern mountains (319.05 g/m^2^) than that in northern (112.35 g/m^2^) and central (126.4 g/m^2^) mountains (*P*<0.05).

BB changed with a wave form and tended to decrease (*R*^2^ = 0.206, *P*>0.05) with larger values appearing in the northern sites of BT and DD as well as the central HY site. Meanwhile, BB in northern mountains (622.62 g/m^2^) was significantly greater than that in the central (431.39 g/m^2^) and southern (437.18 g/m^2^) mountains (*P*<0.05).

TB had no obvious trends (*R*^2^ = 0.018, *P*>0.05) with larger values presenting in the central HY site, the northern BT, and the southern SE site. The TB in central mountains (557.79 g/m^2^) was significantly smaller than that in the northern (734.97 g/m^2^) and southern (756.23 g/m^2^) mountains (*P*<0.05).

#### The spatial distribution of biomass

**Fig 6:**
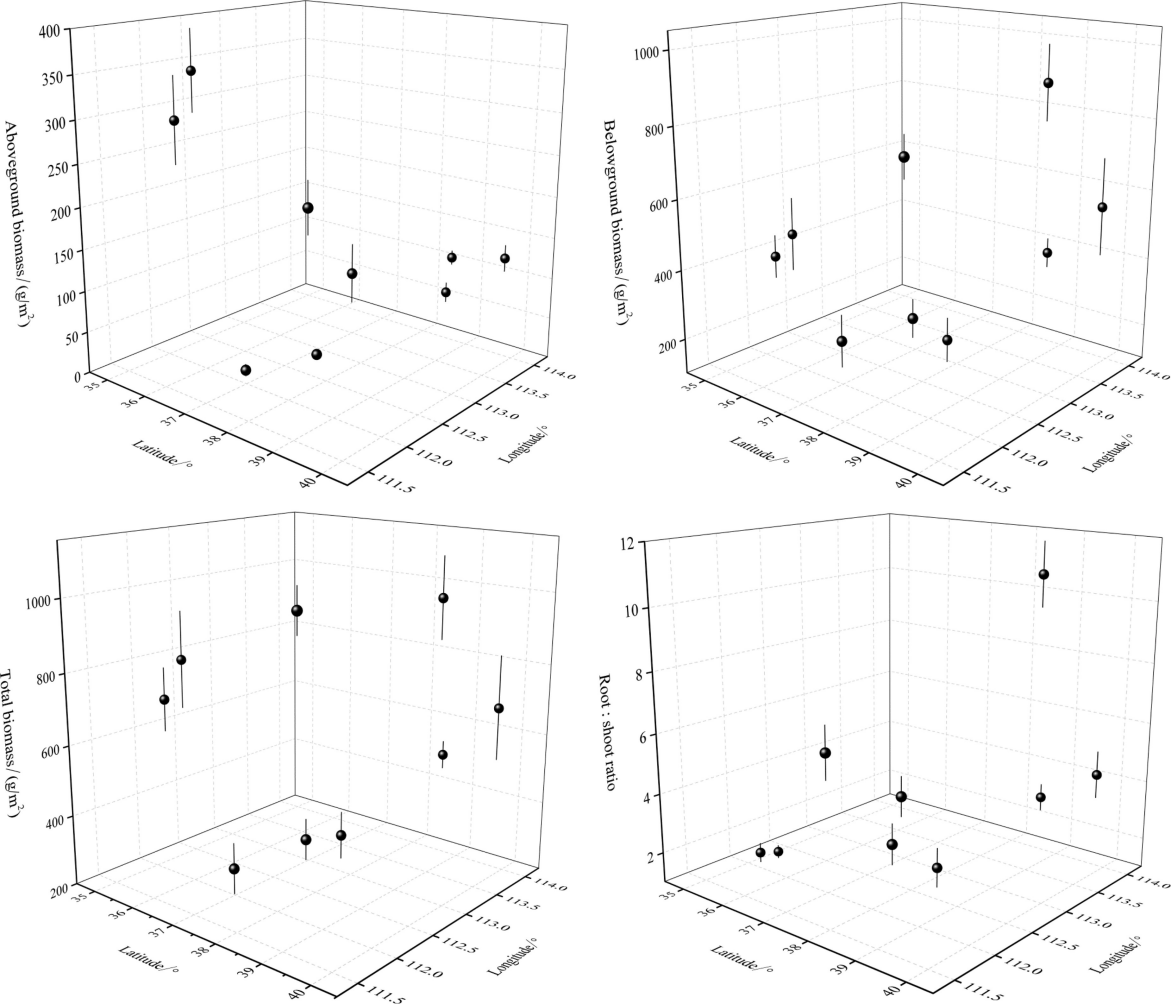
Variations of biomass with latitude and longitude in subalpine meadows. Biomass indices included aboveground, belowground, and total biomass as well as the root:shoot ratio. The data were collected from six quadrats on each of nine mountain sites (54 quadrats in total); means for each mountain site were used in the analysis. Therefore, each index had nine values.

The horizontal spatial distribution of biomass had obvious characteristics in subalpine meadows (Fig 6). Tendencies of AB were apparently stronger with changes in latitude (*R*^2^ = 0.6075) than longitude (*R*^2^ = 0.0677). The BB had slightly smaller tendencies with latitude (*R*^2^ = 0.0691) than longitude (*R*^2^ = 0.1384). Tendencies of TB were also slightly smaller with latitude (*R*^2^ = 0.0187) than longitude (*R*^2^ = 0.0673). That is, changes in AB were more evident along latitudinal gradients, whereas changes in BB were smaller along latitudinal and longitudinal gradients.

**Fig 7:**
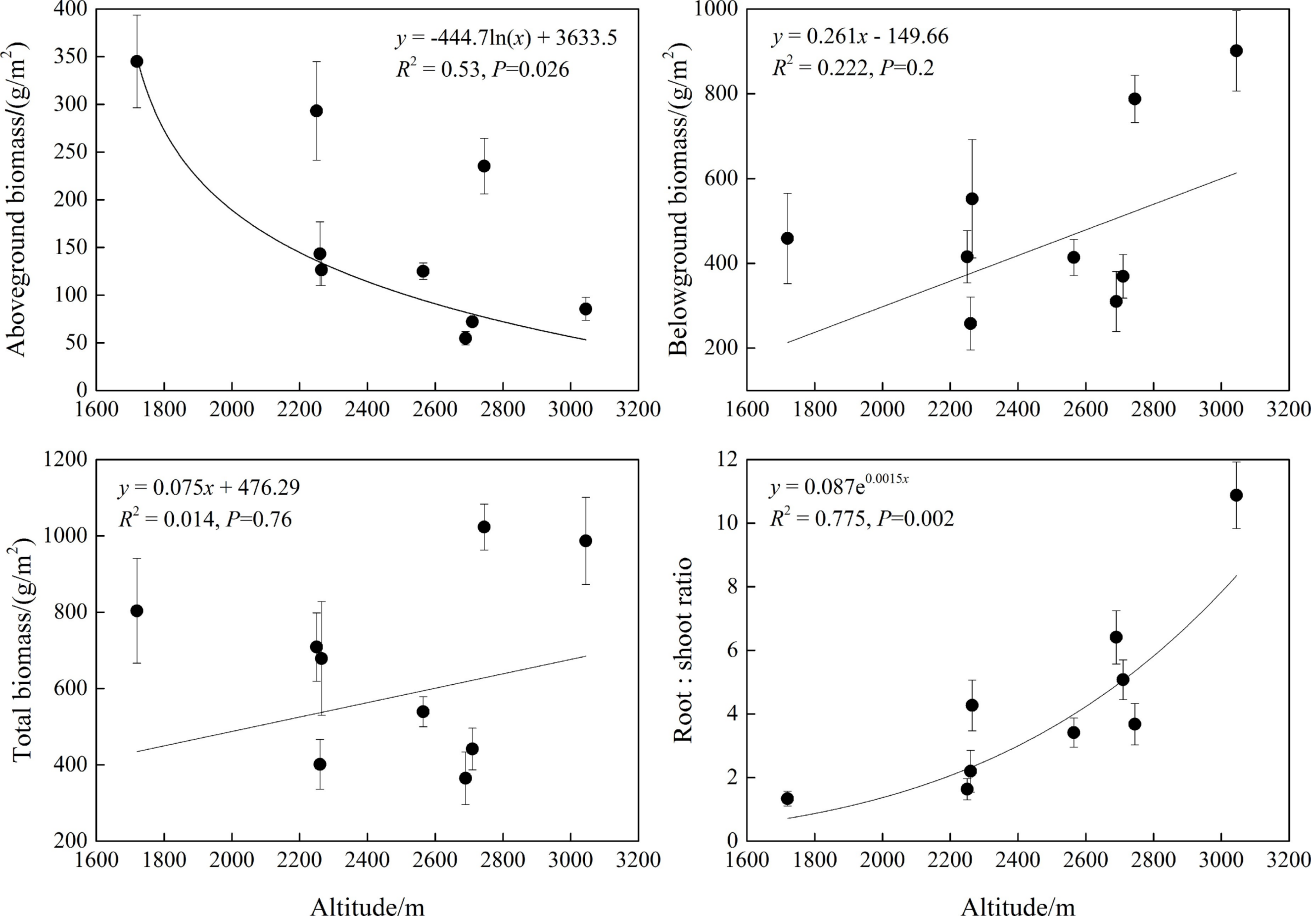
Changes of biomass with elevation in subalpine meadows. The data were collected from six quadrats on each of nine mountain sites (54 quadrats in total); means for each mountain site were used in the analysis. Therefore, each biomass index had nine values.

Along elevational gradients, trends of biomass existed with some differences as well (Fig 7). AB decreased significantly with elevation (*P*<0.05), but the increases in BB and TB were all not significant with increasing elevation (*P*>0.05), illustrating that correlations were stronger between AB and elevation.

#### The variation of biomass allocation relation with different geographical scales

The relation of biomass allocation (R/S) changed in a wave pattern and tended to decrease (*R*^2^ = 0.315, *P*>0.05) with larger values being in the northern BT and central YD and ML sites. The R/S in the southern mountains (1.49) was obviously smaller than that in the northern (6.19) and central (4.34) mountains (*P*<0.05). From DD to DT in the northern mountains, R/S had a tendency of increasing, indicating that more biomass was allocated to belowground plant parts in northern mountains.

With increasing latitude and longitude, R/S tended to increase. Moreover, the R/S obviously became larger with latitude (0.4896) than with longitude (0.0868). That is, more biomass was allocated to belowground plant parts with developmental directions to the north and east in subalpine meadows.

Along elevational gradients, the R/S had significant increasing correlations with increasing elevation (*P*<0.01), indicating that more biomass was also allocated to belowground plant parts with increasing elevation.

### Relationships between species diversity and biomass in subalpine meadows

**Fig 8:**
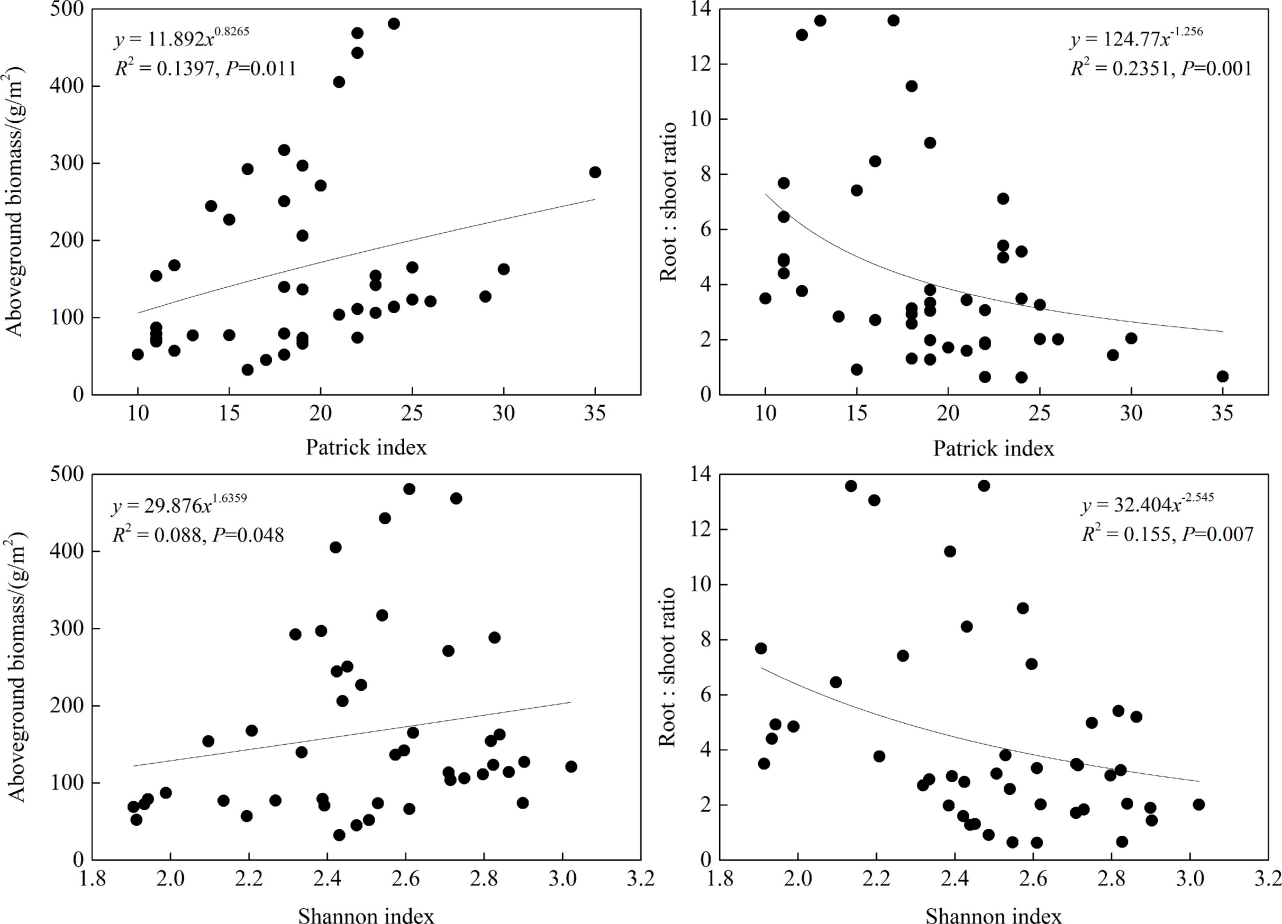
Regression analysis between *α*-diversity and biomass in subalpine meadows. The data were collected from six quadrats on each of nine mountain sites (54 quadrats in total); means for each mountain site were used in the analysis. Therefore, each *α*-diversity and biomass index had 54 values. Significant relationships were selected between *α*-diversity and biomass indices.

We conducted regression analysis between pairs of *α*-diversity and biomass indices to discuss relationships between species diversity and biomass in subalpine meadows (Fig 8). Only *R* and *H* had significant correlations with AB and R/S, respectively, indicating that species diversity had greater effects on AB than on the R/S. AB had significantly positive correlations with *R* and *H* (*P*<0.05), whereas R/S had most significantly negative correlations with *R* and *H* (*P*<0.01); this means that with increasing species diversity, AB increased and more biomass tended to be allocated to aboveground plant parts. In addition, regressive relationships of *R* and *H* conformed to power functions with AB and R/S, respectively, and their power exponents had gaps larger than 1, demonstrating that species diversity had an allometric relationship with biomass in subalpine meadows.

## Discussion

### Spatial distribution of species diversity

Species distribution patterns are outcomes of many ecological processes, which are controlled by species evolution (establishment, migration, and extirpation), variations in geography, and environmental factors (geology, geomorphology, climate, and soil); these then create relatively large discrepancies for patterns of species diversity along geographical gradients for researchers [21, 35, 36]. This primarily occurred in two ways: horizontally and based on elevation. First, in horizontal patterns of distribution, some studies found that species richness had tendencies to decrease with increasing latitude and longitude [30, 37, 38]. Other studies, however, showed that species richness had no significant trends or presented quadratic function variations along latitudinal and longitudinal gradients [39, 40]. Second, in vertical distribution patterns along elevational gradients, some studies indicated that *α*-diversity peaked at central elevations [41–43], whereas other studies showed that *α*-diversity tended to decline gradually with increasing elevation [44] or had no relationships with elevation [34]. Our study found that *α*-diversity had unimodal change patterns with peak values being at high latitude and low longitude in horizontal spaces in subalpine meadows of the Loess Plateau; trends were more distinct along latitudinal gradients, but *α*-diversity was not sensitive to elevation in vertical spaces and tended to decline with increasing elevation.

From research studies on patterns of variation in *β*-diversity along elevational gradients, many scholars also obtained different results. These could be roughly divided into three types. First, *β*-diversity had no regular changes with elevation, and greater values usually appeared in community ecotones [45]. In the second type, *β*-diversity had monotonic decrements with increasing elevation [46, 47]. Last, *β*-diversity rarely changed at low elevation, but sharply increased at high elevations and with increasing elevation [48]. In our research on subalpine meadows, with increasing latitude, longitude, and elevation, the species turnover rate declined and community composition similarity was magnified; therefore, *β*-diversity was diminished, and amplitudes of variation were the greatest along latitudinal gradients, taking second place along elevational gradients, and were smallest along longitudinal gradients.

*γ*-Diversity presented two universal distribution patterns along elevational gradients. One was partial peak in distribution patterns with peak values of different studies appearing in different regions [47, 49] and the other featured negative correlation patterns with linearly decreasing trends [50]. Our study found that total species richness had significant changes with a quadratic function that initially increased and then decreased with longitude and elevation as well as at latitudes of 37.5–40° in subalpine meadows of the Loess Plateau. We found that spatial distributions of *γ*-diversity were in accordance with unimodal change patterns and species distribution was slightly greater in horizontal spaces than with changes in elevation.

Patterns of the distribution of species diversity were closely correlated to scale and environmental factors; in addition, these factors controlled patterns of species diversity that varied tremendously at different scales [6]. Therefore, various scales ought to be sufficiently considered in studies of patterns of species diversity. These scales not only should include scales of environmental gradient, but also should incorporate scales of classification hierarchy [6]. Distribution patterns of species diversity along environmental gradients varied widely among species with various biotypes. For herbaceous communities, these were familiar distribution patterns for *α*-diversity showing unimodal patterns with latitude and reduced trends with increasing elevation. For species with different life-forms, *β*-diversity possessed similar distribution patterns along environmental gradients that decreased with increasing gradients. For *γ*-diversity, it mostly exhibited partial peak distribution patterns with environmental gradients. Hence, distribution patterns of species diversity were different for various vegetation forms and spatial scales. Therefore, many researchers have proposed different theories and hypotheses that can be used to decipher these differences, such as Rapoport’s law, the energy hypothesis, and the mid-domain effect [51, 52].

The horizontal spatial distribution of species diversity approximated unimodal change patterns in subalpine meadows of the Loess Plateau; these distributions were more obvious along latitudinal gradients, which were intimately related to the local natural environment and human activities. The Loess Plateau has climatically severe environments caused by the dry climate, concentrated precipitation, sparse vegetation, and serious soil erosion, as well as the influence of human disturbance such as grazing and tourism. From north to south on the east of the Loess Plateau, the elevation of mountains gradually becomes lower, but human disturbance tends to be enhanced, so that serious human disturbance has negative effects on species diversity at low latitudinal regions. However, in high latitudinal regions, the cold climate slows soil formation and plant growth, where other adverse environments exceed the limitations of tolerance of the majority of species such as intense solar radiation and large differences between day and nighttime temperatures. Therefore, the central latitudinal regions serve as transitional areas with plant species differentiation falling between the above extremes, and these area have relatively greater species diversity [33]. This conclusion verified the advantages of the mid-domain effect.

Regarding the elevation in our study area, the mean elevation was 2472 m in subalpine meadows, but they were mostly between elevations of 2250 m and 2745 m with differences being less than 500 m. This indicates that the elevational differences of the mountains on the east of the Loess Plateau were too small to have apparent effects on local species diversity. However, *α*-diversity of subalpine meadows slightly decreased with increasing elevation, whereas *β*-diversity tended to decease along spatial gradients, demonstrating that species diversity was high in areas with relatively high temperature (S1 Fig). This conclusion agreed perfectly with the environmental energy hypothesis and also provided further proof of the validity of Rapoport’s law [51]. This finding inferred that climates were relatively frigid in high elevational and latitudinal regions and changed more dramatically than climates at low elevational and latitudinal regions, which resulted in broader distribution ranges of plants at high elevational and latitudinal regions. Thus, their species turnover rates between adjoining gradient belts were smaller than in low elevational and latitudinal regions.

### Spatial distribution of biomass

Since the 20th century, additional research on biomass has been conducted on forest plants growing in mountainous areas [53–55]. However, few research studies have addressed biomass allocation for alpine herbaceous plants along spatial gradients; the vast majority of studies have merely been limited to a certain spatial scale such as slope position and elevation with study areas being mostly concentrated on the Qinghai-Tibetan Plateau and the Swiss Alps [41, 56–59]. As a result, research on the distribution and allocation of herbaceous biomass is extremely rare at different spatial scales in other high-elevation and mountainous regions.

Studies on alpine meadows have illustrated that plants allocate more biomass to belowground parts with increasing elevation, and individual plants tended to become dwarfed as one moves from subalpine areas to snow belts; this change leads to decrements in ratios of aboveground plant biomass to belowground biomass and total biomass, respectively [41]. This finding demonstrates that the allocation of plants to sexual reproduction declined and asexual reproduction was augmented with increasing elevation; in addition, R/S increased with decreasing stem biomass and increasing root biomass (especially fine roots with diameters less than 2 mm). This promoted belowground plant parts to acquire sufficient nutrients and to adapt temperatures in order to adapt to extreme environments, such as strong wind, low temperature, and depleted soil in high-elevation mountains [60–62]. As is well-known, subalpine meadows develop in mountains at relatively high elevations, with relatively low micro-habitat temperatures. As trade-offs for meadow plants on resource investments for growth and development, cold environments induced plants to allocate more assimilation substances to belowground organs, especially to underground storage organs, allowing the plants to facilitate the establishment of plants through germination and a resistance of alpine environmental stress; this caused individual plants to produce larger R/S [41].

We concluded that the spatial distribution of aboveground biomass tended to be more obvious at high environmental gradients in subalpine meadows of the Loess Plateau with variations of aboveground biomass being greater along latitudinal gradients and belowground biomass having smaller differences along latitudinal and longitudinal gradients. Toward the north and east in the study area, more biomass was allocated to belowground plant parts and biomass allocation was more evident along latitudinal gradients than longitudinal gradients. From low to high elevation, biomass allocation also tended to emphasize belowground plant parts while the amount of aboveground biomass was significantly reduced, demonstrating that a close correlation existed between aboveground biomass and elevation.

In previous studies, plant biomass in mountainous areas exhibited horizontal variation patterns of increasing from west to east and from north to south [30, 63, 64]. However, the vertical distribution of plant biomass was more complex and assumed either negative correlations [65], unimodal patterns [16], or nonlinear response relationships [53] with variations in elevation. The causes of various patterns of change in biomass are still uncertain along spatial gradients, and a majority of studies show that ratios of water to heat were a primary reason that gave rise to differential distributions of biomass [1, 66, 67]; however, other mechanisms remain unclear. Moreover, plants of different function groups possessed differential responses to changes in environmental factors [68]. Therefore, plants of subalpine meadows allocated more biomass to belowground parts with spatial gradients increasing with elevation in the Loess Plateau. Thus, it is important to explore the influence of environmental factors on a plant community by studying the effects of environmental gradients on species diversity and biomass from the level of plant population.

### Relationship between species diversity and biomass

Relationships between species diversity and biomass can reveal how ecosystems function and can help researchers understand the processes involved in biodiversity as it relates to ecosystem functioning [69–72]. In our research, relationships between species diversity and biomass were in accordance with allometric models in subalpine meadows of the Loess Plateau; biomass allocation tended toward producing aboveground plant parts with increments of species diversity, and thus produced enlargements on aboveground biomass. Researchers have generally acknowledged that biomass was enhanced with increasing species diversity and showed that a power function relationship between them [73]. Results from human-made ecosystems have indicated that systems with the most abundant species produced the greatest biomass, that is, poly-species systems had larger biomass than rare-species systems [8, 74]; experiments in natural grasslands and abandoned lands also illustrated that species diversity had distinct effects on maintaining biomass levels [5, 75]. Nevertheless, this might be caused by sampling effects as well, that is, when more species were selected from a species pool, greater probabilities existed for finding high-biomass species, and then ecosystem productivity increased, which was not induced by increments of species diversity [76, 77]. Under field conditions, because species diversity is influenced by environmental and human disturbance, correlations had complex relationships between species diversity and biomass [68], which was usually manifested in four types: linear relationships [78], nonlinearly unimodal relationships [79], S-type curves [8, 80], and non-correlated relationships [75, 81]. Studies in recent years have principally emphasized the effects of human factors on species diversity, while correlations between species diversity and biomass have not been given enough attention under natural conditions [17, 78, 82, 83].

Furthermore, owing to discrepancies in study scales, projects, and areas, conclusions from studies on correlations also had great differences between species diversity and biomass [84]. For example, in several studies [85–89], (1) topographical disturbances were eliminated by using studies at larger geographical scales, but concrete differences in community diversity were neglected when geographical units had small scales; (2) simulation studies were carried out in homogeneous habitats and artificial communities at small scales, but the influence of stronger spatial heterogeneity caused by enlargements of scale were omitted in studies of diversity and productivity; and (3) in mountainous research studies, scales were focused only on a single space level and lacked systematic research in mountains at overall space hierarchies.

The positive correlation of species diversity with biomass in our study was probably a result that agreed with the biogeographic affinity hypothesis; that is, the ability of species to tolerate climate probably developed under dual effects of Earth’s climates and species evolution in ecological niches [30, 90]. Meanwhile, this result supports our common understanding that high levels of species diversity provide an important way for ecosystems to maintain biomass, that is, greater species diversity realizes accommodation to an environment by providing for species redundancy and functional complementation, and thus sustained the relative stability of productivity in alpine grasslands [30]. Thereby, from a level of plant population in natural conditions, species diversity has important significance to the discussion of spatial distributions and correlations of species diversity and biomass at various levels; this illuminates the internal mechanisms of functional relationships between biodiversity and ecosystems.

## Conclusions

We reported the distribution patterns and correlations of species diversity and biomass at various spatial scales in subalpine meadows on the east of the Loess Plateau in China.

1. *α*-Diversity of subalpine meadows presented unimodal change patterns with smaller values in the central mountains. This was more obvious along latitudinal gradients, but it was not sensitive to the effects of elevation. *β*-Diversity had tendencies to decrease with increasing spatial gradients and amplitudes of variation that were greatest along latitudinal gradients. *γ*-Diversity generally conformed to unimodal change patterns in spatial distribution, which was slightly larger in horizontal spaces than with elevation. The relationship among *α*, *β*, *γ* diversity conformed to the function of *β* = *γ*/*α*.
2. The spatial distribution of biomass in subalpine meadows tended to exhibit high geographical gradients and more biomass was allocated to belowground plant parts with increased spatial gradients. However, responses of aboveground AB to variations in spatial gradient were more sensitive than that of BB. Correlations of species diversity with biomass were in accordance with an allometric model in subalpine meadows; biomass allocation tended to favor belowground plant parts with increasing species diversity, and thus enlarged aboveground biomass.

## Supporting information

S1 Table Abbreviations of indices in this study: *α*-, *β*-, and *γ*-diversity, along with biomass had four, three, one, and four abbreviated indices, respectively. (DOCX)

S1 Fig Temperature data for nine mountain sites analyzed in this study in 2016. In this figure, the upper and lower parts provide the deciphered trends of mean monthly temperature with various months on nine mountains and a significant trend of annual mean temperature on nine mountains (*P*<0.05). Data are from meteorological bureaus of various cities or counties: Guangling County for DD, Wutai County for BT and DT, Ningwu County for ML, Wuzhai County for HY, Yuanping City for YZ, Loufan County for YD, Yuanqu County for SU, and Yangcheng County for SE. Table 1 provides the full names for each mountain with acronyms only used in this figure.
(TIF)
