# Supplementary material for: Multiscale spatial patterns of species diversity and biomass together with their correlations along geographical gradients in subalpine meadows
Source: PLoS One. 2019 Feb 27;14(2):e0211560. doi: 10.1371/journal.pone.0211560 (PMC6392230; doi:10.1371/journal.pone.0211560)
Supplement: S1 Table — (DOCX) [file pone.0211560.s001.docx]

**S1 Table**

| Type | Index | Abbreviation |
| --- | --- | --- |
| *α*-Diversity | Simpson Index | H′ |
|  | Shannon Index | H |
|  | Pielou Index | E |
|  | Patrick Index | R |
| *β*-Diversity | Cody Index | β_C_ |
|  | Sørenson Index | β_S_ |
|  | Bray-Curtis Index | β_B–C_ |
| *γ*-Diversity | Total species richness | S |
| Biomass | Aboveground biomass | AB |
|  | Belowground biomass | BB |
|  | Total biomass | TB |
|  | Root-to-shoot ratio | R/S |
